# Impact of Shunt Placement on CSF Dynamics

**DOI:** 10.3390/biomedicines12010020

**Published:** 2023-12-20

**Authors:** Cyrille Capel, Kimi Owashi, Serge Metanbou, Johann Peltier, Olivier Balédent

**Affiliations:** 1 Department of Neurosurgery, Hospital University Center of Amiens-Picardie, 80054 Amiens, France; peltier.johann@chu-amiens.fr; 2 CHIMERE UR UPJV 7516, Jules Verne University, 80000 Amiens, France; owashi.kimi-ext@chu-amiens.fr (K.O.); olivier.baledent@chu-amiens.fr (O.B.); 3 Image Processing Department, Hospital University Center of Amiens-Picardie, 80054 Amiens, France; 4 Radiology Department, Hospital University Center of Amiens-Picardie, 80054 Amiens, France; metanbou.serge@chu-amiens.fr

**Keywords:** hydrocephalus, hydrodynamic, cerebrospinal fluid, phase-contrast MRI

## Abstract

Background: CSF dynamics are disturbed in chronic hydrocephalus (NPH). We hypothesise that these alterations reflect a disturbance of intracranial compliance. The aim of our study is to investigate the variations in intracranial hydrodynamics in NPH after ventricular shunt surgery. Patients and method: We included 14 patients with definite NPH. All patients improved after ventriculoperitoneal shunting. The patients underwent an analysis of intracranial haemodynamics by phase-contrast MRI (pcMRI) preoperatively, at 6 months postoperatively, and at 1 year postoperatively. We analysed the dynamics of intraventricular CSF at the level of the aqueduct of Sylvius (SV_AQU_) and CSF at the level of the high cervical subarachnoid spaces (SV_CERV_). We calculated the ratio between SV_AQU_ and SV_CERV_, called CSF_RATIO_, which reflects the participation of intraventricular pulsatility in overall intracranial CSF pulsatility. Results: SV_AQU_ significantly (*p* = 0.003) decreased from 240 ± 114 μL/cc to 214 ± 157 μL/cc 6 months after shunt placement. Six months after shunt placement, SV_CERV_ significantly (*p* = 0.007) decreased from 627 ± 229 μL/cc to 557 ± 234 μL/cc. Twelve months after shunt placement, SV_CERV_ continued to significantly (*p* = 0.001) decrease to 496 ± 234 μL/cc. CSF_RATIO_ was not changed by surgery. Conclusions: CSF dynamics are altered by shunt placement and might be a useful marker of the shunt’s effectiveness—especially if pressure values start to rise again. The detection of changes in CSF dynamics would require a reference postoperative pcMRI measurement for each patient.

## 1. Introduction

A preoperative diagnosis of normal-pressure hydrocephalus (NPH) can be a challenge for clinicians. Various clinical scoring systems have been set up for the selection of patients requiring shunt placement and for objective postsurgical follow-up [1,2,3,4]. NPH is treated via the surgical placement of a ventricular shunt; the diagnosis is then made retrospectively, on the basis of the shunt’s effectiveness [1,5]. Various methods for the preoperative diagnosis of NPH have been developed: morphological MRI, clinical response after a cerebrospinal fluid (CSF) tap test, neuropsychological assessments, and analyses of intracranial pressure (ICP) during infusion. The latter technique provides information on the barometric properties of the craniospinal system. Phase-contrast MRI (pcMRI) enables the non-invasive analysis of craniospinal haemodynamics and CSF dynamics during the cardiac cycle (cc).

After shunt placement, there are hydrodynamic changes in terms of ICP analysis during infusion tests [6]. ICP exhibits pulsatility during the cc, which is a result of craniospinal haemodynamic and hydrodynamic interactions [7,8]. This aspect can be analysed non-invasively using pcMRI [9].

Under normal conditions, pulsatile CSF flows are driven by cerebral blood inflows and outflows. Arterial blood inflow varies throughout the cc, with a systolic arterial peak and a diastolic trough [8]. Venous blood outflow is less pulsatile, and peak systolic flow is lower and later than peak arterial blood inflow [8]. The lack of synchronisation between blood inflow and blood outflow results in changes in intracranial blood volume during the cc. The CSF moves to the spinal subarachnoid spaces through the foramen magnum during systole and returns to the intracranial compartment during diastole [8,9,10,11]. This CSF volume moves back and forth and so is referred to as the cervical stroke volume (SV_CERV_). SV_CERV_ measured in the subarachnoid space is about 450 μL per cc [8]. Aqueductal CSF SV (SV_AQU_) corresponds to the volume of CSF displaced into the aqueduct of Sylvius during a single cc and is around 10 times smaller than SV_CERV_. pcMRI is the only quantitative, non-invasive tool for the investigation of neurofluid dynamics during the cc.

Patients with NPH present with CSF flow disorders, including intraventricular CSF hyperpulsatility [12,13,14,15,16]. SV_AQU_ increases as NPH progresses [16]. However, no evidence of cervical CSF flow disorders has been found in patients with hydrocephalus [12]. In healthy individuals, intracranial CSF compliance is mainly due to CSF subarachnoid pulsations [8,17]. In people with NPH, intraventricular CSF pulsatility has a major role in intracranial compliance and in intracranial pressure damping during vascular expansion [12]. Some researchers have found that neither SV_CERV_ nor SV_AQU_ is correlated with ventricular volume [18]. Nevertheless, this point is subject to debate [19,20].

The value of pcMRI for the diagnosis of chronic hydrocephalus is also subject to debate. In the 1990s, it was suggested that SV_AQU_ was a diagnostic marker for NPH [21,22,23]. Nevertheless, this notion was intensely debated in the 2000s and 2010s [14,24]. At present, SV_AQU_ is not considered to be a diagnostic marker. CSF dynamics are closely related to cerebral haemodynamics. An overall analysis of craniospinal haemodynamics and hydrodynamics (i.e., neurofluid interactions) is therefore necessary [12,17,25].

As mentioned above, NPH is treated via shunt placement [26]. In a pcMRI analysis, Scollato et al. demonstrated that SV_AQU_ decreases after shunt placement [27] in patients with clinical improvement and also in those without—although the decrease was greater in the former. Ringstad et al. reached a similar conclusion [19]. Other pcMRI studies have assessed postsurgery changes in CSF flow within the aqueduct but did not measure the SV [28]. To the best of our knowledge, postsurgery changes in overall, intraventricular, and subarachnoid CSF dynamics in people with NPH have not previously been studied. Hence, the objective of the present study was to compare CSF dynamics before and after shunt placement.

## 2. Materials and Methods

### 2.1. Patients

We retrospectively included patients with a diagnosis of NPH, as confirmed by clinical improvement after the ventriculoperitoneal placement of a flow-regulated shunt (OSVII, Integra Lifesciences^®^, Sophia-Antipolis, France). Clinical status was evaluated on the idiopathic NPH grading scale [4] before shunt placement and 6 months after shunt placement. We analysed gait disorders, the living situation, and urinary continence. An improvement was defined as an increase in the iNPH score of 10% or more.

All the patients underwent pcMRI before shunt placement and then 6 and 12 months after shunt placement. Patients with no improvements on the iNPH scale were excluded.

### 2.2. pcMRI Acquisition

**Figure 1:**
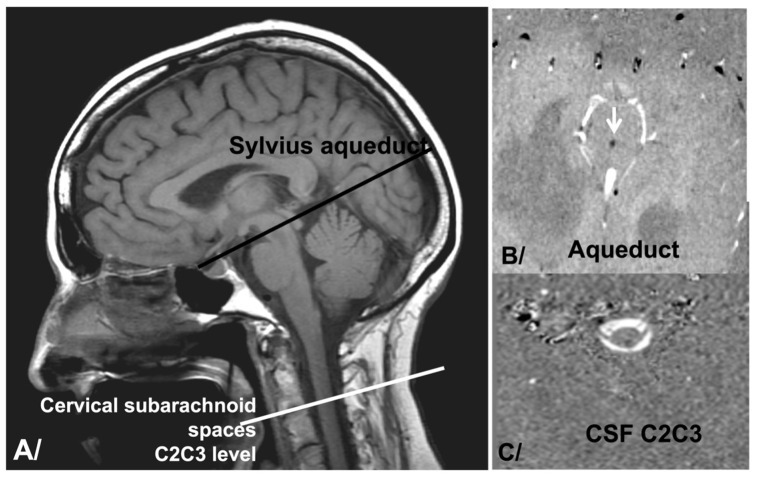
Slice plan positioning. (**A**) Positioning of pcMRI slice plane on sagittal T1 images at the level of the sylvian aqueduct to analyse intraventricular CSF dynamics and at the level of C2C3 intervertebral disc to analyse cervical subarachnoid CSF dynamics. (**B**) pcMRI acquisition at the level of the sylvian aqueduct (arrow). It appears in black due to the direction of the flow from the fourth ventricle to the third ventricle. (**C**) pcMRI acquisition of cervical subarachnoid spaces at the level of the C2C3 intervertebral disc (arrow). These spaces have the form of a white crown. It appears in white due to the craniocaudal direction of the flow.

In our diagnostic work-up, we added pcMRI sequences to conventional morphological sequences. Brain MRI was performed on a 3T machine (Philips Achieva: maximum gradient: 80 mT/m; rate of gradient increase: 120 mT m^−1^ ms^−1^) with the following imaging parameters: repetition time (TR): as low as possible, depending on the heart rate; echo time (TE) as minimum; field-of-view: 140 cm; matrix: 256 × 256; and slice thickness: 5 mm. Cardiac gating was achieved via a plethysmograph positioned on the finger. A total of 32 ccs were retrospectively reconstructed, and a single mean value was produced. We quantified CSF oscillations through the mesencephalic aqueduct and through the spinal subarachnoid spaces at the C2C3 intervertebral disc (Figure 1). For the examinations 6 and 12 months after shunt placement, the slice plane’s position was adjusted to match that used before shunt placement. The use of CSF dynamics within the aqueduct is common in the literature. Moreover, this location enables the analysis of intraventricular CSF dynamics, which do not behave in the same way as subarachnoid CSF. Analysis of the CSF at the level of the high cervical spine is the result of the overall dynamics of intracranial and intraventricular CSF. It bears witness to intracranial strain. Some authors have suggested that it is the mobile compliance of the craniospinal system that regulates intracranial pressure. We therefore proposed an analysis of these 2 regions.

The velocity encoding parameter (VENC) was adjusted so that it was as close as possible to the fluid’s expected maximum velocity. For CSF flow, we selected a value of 5 cm/s for the neck and a value of 10 cm/sec at the aqueduct. If only few pixels required aliasing, we applied an automatic aliasing correction algorithm during postprocessing [7]. If many pixels required aliasing, the pcMRI acquisition was repeated with twice the previous VENC. The acquisition lasted for 2 min.

### 2.3. Data Analysis

pcMRI acquisition data were analysed using in-house software (Flow 2.0—March 2021). The aqueduct and spinal subarachnoid areas were detected automatically by a dedicated segmentation algorithm [7].

CSF segmentation involved the creation of a new parametric image and then the application of a threshold to the new image. Firstly, to speed up the data processing steps, we quickly drew a rectangular region of interest (ROI) around the CSF spaces to be segmented. This ROI was then applied to the 32 timeframes of pcMRI phases, where the intensity of each phase pixel represents the velocity of the voxel. We produced a new parametric image by applying a fast Fourier transform to the time-domain matrix. The intensity of each pixel in the parametric image corresponds to the fundamental frequency of the person’s heart rate. Pixel intensity in the new image is higher for voxels exhibiting cardiac periodicity (such as the CSF) than for voxels which are not synchronised with the heart rate. We next extracted all the pixels with a velocity above a visually selected threshold; by choosing the right threshold value, we were able to identify tissue with a smaller fundamental component than CSF pulses (which have a large fundamental component). We have described this algorithm’s reproducibility and accuracy elsewhere [8].

The CSF flow dynamic curve was calculated and reconstructed with 32 points in order to represent typical CSF flow during the cc. To correct for eddy currents and calibrate the velocity, we selected a background area located close to our ROI. The software (Flow 2.0—March 2021) automatically calculated the CSF SVs in mL per cc [11].

Lastly, we defined the CSF SV ratio as SV_AQU_ × 100/SV_CERV_; this corresponds to the intraventricular CSF’s contribution to the movement of the intracranial subarachnoid CSF into spinal subarachnoid spaces through the foramen magnum.

### 2.4. Statistical Analysis

We used paired Wilcoxon’s tests to compare SVs before and after shunting. We also applied Student’s *t*-test, after checking that the data were normally distributed. The threshold for statistical significance was set to *p* < 0.05 in all cases.

### 2.5. IRB/Ethics

The study protocol was approved by the local independent ethics committee (*CPP Nord Ouest II*, Amiens, France; reference: PI2023_843_0065). In line with French legislation on retrospective observational studies of clinical practice, patient consent was not required.

## 3. Results

Sixteen patients were included prospectively. However, two patients were lost to follow-up and so were excluded. Hence, 14 patients with confirmed NPH (mean ± standard deviation (SD) age: 71.6 ± 8.84; range: 52.2–86.5) were included in the final analysis.

### 3.1. CSF Dynamics before Shunting

Mean SV_AQU_ was 240 ± 114 μL/cc (Table 1), mean SV_CERV_ was 627 ± 229 μL/cc, and the CSF SV ratio was 40 ± 20%.

### 3.2. CSF Dynamics Evolution after Shunt Placement

**Figure 2:**
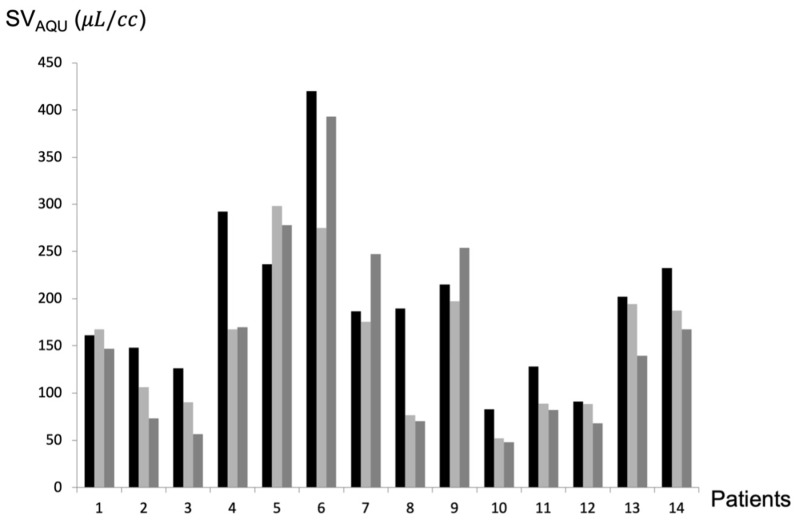
Aqueductal CSF stroke volume (SV_AQU_) evolution after shunt. SV_AQU_ was measured for each patient before surgery (black line), 6 months after surgery (light grey), and 1 year after surgery (dark grey). SV_AQU_ decreased after shunting, except for patients 1, 5, 7, and 9. cc: cardiac cycle.

Six months after shunt placement, mean SV_AQU_ had decreased significantly (*p* = 0.03 in Wilcoxon’s test; *p* = 0.04 in Student’s *t*-test) from 240 ± 114 μL/cc to 214 ± 157 μL/cc. Twelve months after shunt placement, SV_AQU_ continued to decrease significantly (*p* = 0.03 in Wilcoxon’s test; *p* = 0.03 in Student’s *t*-test) to 193 ± 145 μL/cc. In two patients, SV_AQU_ increased. (Figure 2). The mean ± SD (range) percentage change in SV_AQU_ was −20.34% ± 23.87 (+26.14%–−59.55%) at 6 months and −25.85% ± 29.26 (+32.30%–−62.82%) at 12 months.

**Figure 3:**
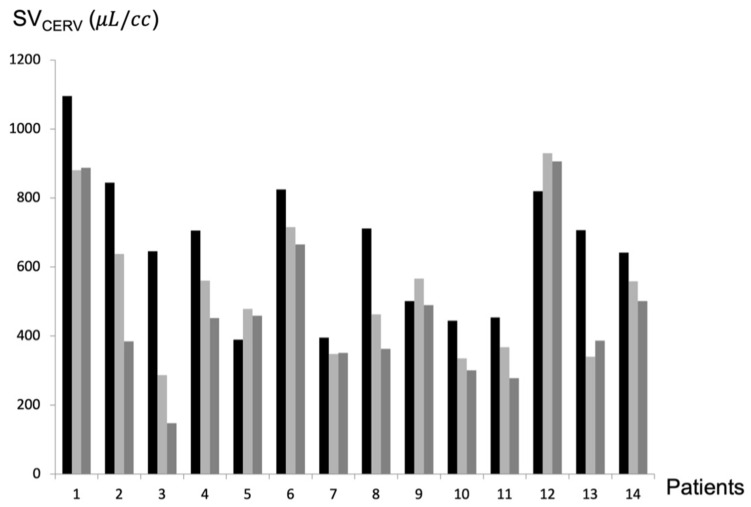
Cervical CSF stroke volume (SV_CERV_) evolution after shunt. SV_CERV_ was measured for each patient before surgery (black line), 6 months after surgery (light grey), and 1 year after surgery (dark grey). SV_CERV_ decreased after shunting, except for patients 9 and 12. cc: cardiac cycle.

Six months after shunt placement, mean SV_CERV_ had decreased significantly (*p* = 0.008 in Wilcoxon’s test; *p* = 0.02 in Student’s *t*-test) from 627 ± 229 μL/cc to 557 ± 234 μL/cc. Twelve months after shunt placement, SV_CERV_ continue to decrease significantly (*p* = 0.005 in Wilcoxon’s test; *p* = 0.003 in Student’s *t*-test) to 496 ± 234 μL/cc (Figure 3). In two patients, SV_CERV_ increased. The mean ± SD (range) percentage change in SV_CERV_ was +19.91% ± 22.61 (+23.02%–−55.47%) at 6 months and 29.46% ± 27.08 (max: +17.98%–−77.05%) at 12 months.

**Figure 4:**
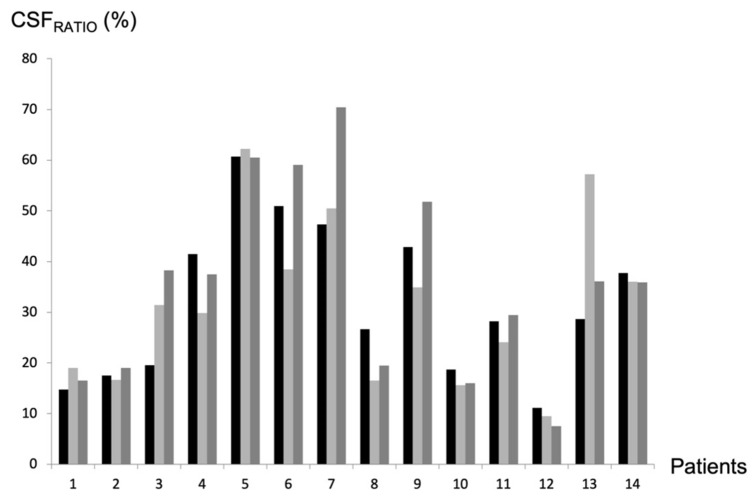
CSF_RATIO_ evolution after shunt placement. CSF_RATIO_ was calculated as follows: $CSFRATIO=\frac{aqueductal\;CSF\;stroke\;volume}{cervical\;CSF\;stroke\;volume}\times 100$. It was translated into percentages. It reflects the participation of intraventricular CSF in overall intracranial CSF pulsatility during a cardiac cycle. CSF_RATIO_ was measured for each patient before surgery (black line), 6 months after surgery (light grey), and 1 year after surgery (dark grey).

Mean CSF_RATIO_ did not change significantly after shunt placement (Figure 4), with a value of 40 ± 20% before surgery, 40 ± 27% 6 months after surgery (*p* = 0.94 in Wilcoxon’s test;; *p* = 0.724 in Student’s *t*-test), and 42 ± 32% 12 months after surgery (*p* = 0.42 in Wilcoxon’s test; *p* = 0.2 in Student’s *t*-test).

All results are summarised in Table 1.

## 4. Discussion

Our analysis of CSF dynamics before and after shunt placement revealed early and late changes—mainly in the cervical subarachnoid spaces.

### 4.1. Preoperative Intraventricular CSF Dynamics (Aqueductal Stroke Volume)

In line with the literature data, we observed intraventricular CSF hyperpulsatility in our patient population: in an earlier study, we found mean SV_AQU_ values of 196 ± 100 μL/cc in people with NPH and 51 ± 25 μL/cc in a control group [12]. Bradley [29] has suggested that intraventricular pulsatility is linked to centripetal strain during vascular expansion, which increases intraventricular CSF flushing to the subarachnoid spaces during systole. Of course, the decrease in ICP during diastole (due to cerebral venous aspiration by the heart) prompts the ventricles to fill with CSF.

Measurements of ICP during an infusion test showed an increase in resistance to CSF outflow (R_out_) in NPH [30,31]. R_out_ reflects the resistance to CSF flow from the production sites to the resorption sites. Impaired CSF flow within the intracranial subarachnoid spaces can lead to impaired compliance and an increase in R_out_. This can be compensated for by the pulsatility of intraventricular CSF. This observation indicates that intraventricular hyperpulsatility results from low CSF pulsatility in the intracranial subarachnoid spaces, which balances vascular blood expansion during the cc: an equivalent volume of intracranial CSF must flow into the spinal canal. Under normal conditions, 90% of this CSF volume arrives rapidly from the intracranial subarachnoid spaces, while only a very small proportion of ventricular CSF flows into the spinal canal.

As mentioned above, our results confirmed that SV_AQU_ is abnormally high in people with NPH. This might be due to greater CSF flow resistance in the intracranial arachnoid spaces, which would limit the free flow of CSF into this compartment. Alternatively, ICP tissue compression might be redistributed during vascular systolic expansion, with a shift from a centrifugal flow to a centripetal flow in the ventricles. Consequently, the intraventricular CSF contributes to intracranial compliance.

### 4.2. Preoperative Global CSF Dynamics (Aqueductal and Cervical Stroke Volumes)

It has been reported that CSF pulsatility in the cervical subarachnoid spaces is slightly higher (by 30%) in healthy young adults than in healthy older adults [32]. Likewise, CSF_RATIO_ is slightly higher in healthy young adults than in healthy older adults [32]. CSF_RATIO_ reflects the contribution of intraventricular CSF flow to cervical CSF flow and to overall intracranial mobile compliance. According to the literature, CSF_RATIO_ is around 10% in healthy adults and around 50% in adults with NPH [12]. With each cc and the associated variations in intracranial vascular volume, the CSF oscillates between the intracranial and spinal subarachnoid spaces. This phenomenon (i.e., mobile compliance) compensates for variations in intracranial volume [9]. Mobile compliance can be measured as the cervical CSF SV using pcMRI. Cervical CSF pulsation reflects overall intracranial compliance. Cervical CSF SV results from the pulsatility of the intracranial subarachnoid and intraventricular CSF flows. CSF_RATIO_ represents the contribution of intraventricular CSF pulsatility to these overall dynamics and thus to mobile compliance. In people with NPH, intraventricular CSF and subarachnoid CSF contribute equally to compensate for vascular expansion. In a control population, subarachnoid CSF contributes predominantly to this process. This observation suggests that resistance to CSF flow into the intracranial subarachnoid spaces increases (e.g., due to the presence of the arachnoid membrane).

### 4.3. Impact of Shunt Placement on Intraventricular CSF Dynamics

Six months after shunt placement, we observed a very small but statistically significant decrease (relative to measurements before surgery) in SV_AQU_. This finding is consistent with the literature data [19,27]. Scollato et al. [27] observed that SV_AQU_ decreased to near-normal values (i.e., by almost 100%) after shunt placement. In our study, the mean decreases were around 20% at 6 months and around 25% at 12 months. The decrease might be influenced by the type of shunt used. Scollato et al.’s study population had pressure-regulated shunts, whereas our population had flow-regulated shunts. Ringstad et al. reported a 32% decrease in SV_AQU_ 12 months after surgery, and the decrease was greater for flow-regulated shunts than for pressure-regulating shunts [19].

A pressure-regulating shunt operates when the ICP exceeds the opening pressure. This opening is temporary and may not even occur in cases of low-pressure hydrocephalus. Flow-regulating shunts provide an alternative drainage pathway, which leads to continuous depletion and an increase in overall intracranial compliance over a longer timescale (i.e., beyond the cc). On the timescale of the cc, flow-regulating shunts maintain a fixed flow rate within a physiological pressure range. A pressure-regulated shunt may operate intermittently during a cc, resulting in ICP variations. As a result, a pressure-regulated shunt can allow variable compliance over a cc and can dampen intraventricular CSF dynamics more consistently than a flow-regulated shunt. Indeed, in a pressure-regulated shunt, flow is a direct function of the position (Trendelenburg or reverse Trendelenburg) as well as the pressure gradient upstream and downstream of the shunt.

We observed small but significant decreases in SV_AQU_ 6 and 12 months after shunt placement. Scollato et al. observed decreases of up to 18% in the first month after shunt placement [27]. Some of Scollato et al.’s patients showed an increase in SV_AQU_ after shunt placement, even though all experienced a clinical improvement. In the case of pressure-regulated shunts, this observation was thought to reflect an excessively high opening pressure setting [27]. In our study, all the implanted shunts were flow-regulated. Conceptually, however, shunts are palliative treatments for conditions whose underlying physiological mechanisms are not known. Therefore, shunts can produce clinical improvements without necessarily addressing the underlying hydrodynamics, and their effectiveness can vary from one patient to another as a function of the aetiology of NPH. The exact cause of idiopathic NPH is not known. At the Hydrocephalus 2023 congress, many experts suggested that the terminology of NPH should be revised to match our evolving physiological knowledge [33]. At present, there is no consensus on the aetiology of impairments in CSF pulsatility [12,19,25,32]. Furthermore, the physiology of CSF is complex and multifaceted; each new investigation reveals inter-individual differences, making it difficult to establish a consensus. Analyses of the overall haemodynamics and hydrodynamics of people with NPH reveal various impairments, such as vascular changes [34] and disruptions in spinal [12], intraventricular [12,13,14], and subarachnoid flows. Compliance of the craniospinal system might be impaired at various sites, with differences between individuals. All these variations contribute to a single nosological entity: NPH.

### 4.4. Impact of Shunt Placement on Cervical CSF Dynamics

We observed a slight decrease in cervical CSF dynamics (SV_CERV_) after surgery, which suggests a reduction in intracranial strain. Cervical subarachnoid CSF is influenced by intracranial strain during the cc, due to vascular expansion. During a cc, vascular volume varies because the arterial blood input is not immediately compensated for by the venous blood output. The intracranial space is limited, and the CSF serves as a mobile compliance mechanism for vascular volume changes by flushing through the foramen magnum. SV_CERV_ corresponds to a combination of CSF pulsations in the intracranial subarachnoid space and in the intraventricular area. A decrease in SV_AQU_ is consistent with a reduction in SV_CERV_. However, SV_CERV_ appears to decrease over a long period.

### 4.5. Impact of Shunt Placement on Global Hydrodynamics

CSF_RATIO_ did not change significantly after shunt placement. This ratio reflects intraventricular CSF’s compensation for vascular expansion. Vascular expansion relies on mobile compliance provided by the CSF in both the intracranial subarachnoid spaces and the intraventricular compartment. In control individuals, CSF_RATIO_ is approximately 10%. In people with NPH, 40% to 50% of the CSF flowing through cervical subarachnoid spaces originates in the intraventricular compartment [12]. This phenomenon may be linked to changes in intracranial subarachnoid space flow [12] or (as suggested by Bradley [29]) centripetal strain within the cranium.

ICP monitoring during infusion studies has revealed an increase in resistance to CSF outflow from production sites to resorption sites [30,35,36,37]. After shunt placement, ICP and resistance to CSF outflow decrease, whereas the compensatory reserve increases; these signs indicate an improvement in CSF circulation and in resorption capacity [6]. ICP monitoring and infusion studies provide information on CSF dynamics and pressure-volume adaptations after surgery [6]. The ICP exhibits pulsatility during the cc. This pulsatility is a result of craniospinal haemodynamic and hydrodynamic interactions [7,8]. This aspect can be analysed non-invasively using pcMRI [9]. Some authors propose a non-invasive measurement of ICP based on measurements of the CSF and blood flows during a cardiac cycle from pcMRI [7]. This has not been validated by comparative studies between invasive and non-invasive measurements in a patient population.

### 4.6. Perspectives

Our study opens up a new field of exploration of chronic hydrocephalus, both physiologically and clinically. Indeed, from a physiological point of view, this study shows that shunt surgery for chronic hydrocephalus leads to clinical improvement, but the effect on the physiology of CSF flows is not constant. In some cases, there is a decrease in intraventricular or subarachnoid CSF pulsatility without returning close to the norms defined in control populations. Nonetheless, there is a change in CSF dynamics that needs to be considered. It may therefore be advisable to perform MRI with phase-contrast sequences to obtain a reference hydrodynamic balance. A change in baseline hydrodynamics could be a non-invasive marker of shunt dysfunction.

## 5. Conclusions

CSF dynamics are altered by shunt placement and might be a useful marker of the shunt’s effectiveness—especially if pressure values start to rise again. The detection of changes in CSF dynamics would require a reference postoperative pcMRI measurement for each patient.

## Figures and Tables

**Table 1:** Craniospinal hydrodynamic evolution after surgery.

	Preoperative PCMRI (T1)	PCMRI 6 Months after Surgery (T2)	PCMRI 12 Months after Surgery (T3)	*p* (Comparison of T1 and T2)	*p* (Comparison of T2 and T3)	*p* (Comparison of T1 and T3)
SV_AQU_	240 ± 114 μL/cc	214 ± 157 μL/cc	193 ± 145 μL/cc	0.003	0.12	0.001
SV_CERV_	627 ± 229 μL/cc	557 ± 234 μL/cc	496 ± 234 μL/cc	0.007	0.001	0.001
CSF_RATIO_	40 ± 20%	40 ± 27%	42 ± 32%	0.52	0.09	0.12

SV_AQU_: stroke volume of intraventricular CSF measured at the level of the sylvian aqueduct; SV_CERV_: stroke volume of subarachnoid CSF measured at the level of the C2C3 intervertebral disc; CSF_RATIO_ = SV_AQU_ × 100/SV_CERV_; T1: preoperative phase-contrast MRI (pcMRI); T2: pcMRI 6 months after shunting; T3: pcMRI 1 year after shunting.

## Data Availability

Data are available up to 9 months after acceptance of the ethical agreement from the 6 April 2023.
